# Social Functioning in Children with Cerebral Palsy with and Without Cerebral Visual Impairment: A Multidimensional Analysis

**DOI:** 10.3390/children13101310

**Published:** 2026-09-26

**Authors:** Mustafa Cemali, Sümeyye Belhan Çelik, Ayşe İlayda Mutlu

**Affiliations:** 1 Department of Occupational Therapy, Faculty of Health Sciences, Trakya University, 22030 Edirne, Türkiye; 2 Department of Occupational Therapy, Hamidiye Faculty of Health Sciences, University of Health Sciences Türkiye, 34668 Istanbul, Türkiye; 3 Department of Speech and Language Therapy, Faculty of Health Sciences, Başkent University, 06790 Ankara, Türkiye

**Keywords:** cerebral palsy, cerebral visual impairment, social functioning, social communication, child

## Abstract

**Highlights:**

**What are the main findings?**

- Cerebral visual impairment status remained independently associated with lower social functioning after age, sex, gross motor function, manual ability, speech performance, and SCQ total score were considered in the hierarchical regression model.
- Gross motor function, speech performance, and SCQ total score were also independently associated with social functioning in children with cerebral palsy.

**What are the implications of the main findings?**

- Social functioning in children with cerebral palsy should be evaluated within a multidimensional framework that considers visual, motor, speech, and SCQ-assessed social communication and behavioral characteristics.
- Assessment of children with cerebral palsy and cerebral visual impairment should extend beyond motor and visual functions to include speech, SCQ-assessed social communication and behavioral characteristics, and social functioning in daily life.

**Abstract:**

**Objective:** This study aimed to compare social functioning and social communication characteristics between children with CP and CVI and children with CP without CVI, and to identify demographic, clinical, and functional factors independently associated with social functioning. **Methods:** This observational cross-sectional study included 70 children with CP aged 48–84 months, including 35 children with CP and CVI and 35 children with CP without CVI. Gross motor function was assessed using the Gross Motor Function Classification System (GMFCS), manual ability using the Manual Ability Classification System (MACS), speech performance using the Viking Speech Scale (VSS), social communication using the Social Communication Questionnaire (SCQ), and social functioning using the Social Function domain of the Pediatric Evaluation of Disability Inventory (PEDI). Between-group comparisons were performed, and hierarchical multiple linear regression analysis was conducted to identify factors independently associated with social functioning. **Results:** Significant between-group differences were found in the distributions of GMFCS (*p* = 0.007), MACS (*p* = 0.043), and VSS (*p* = 0.027) levels. Children with CP and CVI had lower PEDI Social Function scores and higher SCQ total scores than children with CP without CVI (*p* < 0.001). Across all SCQ domains, including reciprocal social interaction, communication, and restricted, repetitive, and stereotyped behaviors, children with CP and CVI showed higher scores (all *p* < 0.001). In the hierarchical regression analysis, the final model explained 96.7% of the variance in social functioning (R^2^ = 0.967; adjusted R^2^ = 0.963; *p* < 0.001). Age (β = 0.089; *p* = 0.008), CVI status (β = −0.295; *p* < 0.001), GMFCS level (β = −0.270; *p* < 0.001), VSS level (β = −0.211; *p* < 0.001), and SCQ total score (β = −0.272; *p* < 0.001) were independently associated with social functioning. **Conclusions:** Children with CP and CVI had lower social functioning and higher scores for SCQ-assessed social communication and behavioral characteristics than children with CP without CVI. The independent associations of CVI status, gross motor function, speech performance, and SCQ total score with social functioning highlight the importance of a multidimensional approach to evaluating social functioning in children with CP.

## 1. Introduction

Cerebral palsy (CP) encompasses a group of permanent movement and posture disorders that result in activity limitations following disturbances in the developing fetal or infant brain [1]. Although CP is primarily characterized by motor impairment, sensory, perceptual, cognitive, communication, and visual impairments are also frequently present [1,2]. Given this multidimensional clinical presentation, a comprehensive functional profile of children with CP requires consideration of gross motor function, manual ability, communication, and visual function together [3]. Visual impairments represent an important component of this profile, and cerebral visual impairment (CVI) is one of the visual disorders frequently observed in children with CP. The prevalence of CVI among children with CP has been reported to reach approximately 60–70%, although reported rates vary depending on the diagnostic criteria used and the population evaluated [4,5]. CVI is characterized by visual dysfunction arising from abnormalities affecting the retrogeniculate visual pathways, visual cortex, and/or visual association areas, without major ocular pathology sufficient to explain the visual difficulties [4,5,6].

Cerebral visual impairment (CVI) may affect how children perceive, process, and use visual information in everyday activities. Its clinical manifestations can involve difficulties with visual attention and perception, scanning and visual search, recognition of objects and faces, visually guided movement, and the interpretation of complex visual environments [5,6,7,8]. In children with CVI, basic visual and oculomotor functions have been reported to be associated with neuropsychological and cognitive–visual characteristics, highlighting the importance of evaluating visual function within a comprehensive functional profile [7]. Visual searching may also be challenging for children with CVI, particularly when they need to locate objects or familiar people within crowded or visually complex environments [8]. These difficulties may hinder a child’s ability to direct attention toward people and socially meaningful stimuli, distinguish faces and facial expressions, and use visual cues during social interactions. However, social functioning in the daily lives of children with CP may be associated not only with visual characteristics but also with gross motor function, manual ability, speech performance, and communication characteristics. Indeed, social functioning in children with CP has been reported to develop with age and differ according to communication function level, while motor and communication abilities have also been associated with social functioning [9,10,11].

Previous studies comparing children with CP and CVI and children with CP without CVI have investigated various developmental and functional domains. Studies examining sensory and motor functions in children with CP and CVI have highlighted the importance of considering different sensory and functional domains alongside visual characteristics [12]. Studies involving children aged 3–6 years have shown that children with CP and CVI differ from children with CP without CVI and typically developing children in terms of sensory processing, behavioral characteristics, and feeding behaviors, and that sensory processing characteristics are associated with behaviors related to daily life [13,14]. Focusing specifically on social development, Kılıç et al. [15] reported lower social competence in children with CP and CVI aged 12–36 months than in children with CP without CVI and typically developing children. However, that study focused on infancy and early childhood and specifically examined social competence. To our knowledge, no study has compared social functioning in daily life between children with CP and CVI and children with CP without CVI while simultaneously examining the independent associations of CVI status, gross motor function, manual ability, SCQ-assessed social communication and behavioral characteristics with social functioning. Therefore, this study aimed to compare gross motor function, manual ability, speech performance, social communication, and social functioning between children with CP and CVI and children with CP without CVI, and to identify factors independently associated with social functioning using a hierarchical approach.

## 2. Materials and Methods

### 2.1. Study Design

This observational cross-sectional study was conducted between April 2024 and April 2026 at a rehabilitation center providing special education and rehabilitation services for children with CP in Ankara, Türkiye. The study protocol was approved by the Lokman Hekim University Scientific Research Ethics Committee (Decision No: 2024/59; Decision Date: 23 February 2024). The study was conducted in accordance with the principles of the Declaration of Helsinki, and written informed consent was obtained from the parents/legal guardians of all children prior to participation.

Two independent groups of children with CP aged 4–7 years were included: children with CP and CVI and children with CP without CVI. In the comparative component of the study, gross motor function, manual ability, speech performance, social communication characteristics, and social functioning were compared between the two groups. In addition, hierarchical multiple linear regression analysis was performed to identify factors independently associated with social functioning and to determine the incremental contribution of these factors to the explained variance in social functioning. In the hierarchical model, social functioning was treated as the dependent variable, while demographic characteristics, CVI status, gross motor function, manual ability, speech performance, and social communication characteristics were entered into the model in sequential blocks based on a theoretically and clinically informed order. The study was reported in accordance with the recommendations of the Strengthening the Reporting of Observational Studies in Epidemiology (STROBE) statement (Supplementary File S1) for cross-sectional studies [16].

### 2.2. Participants

An a priori power analysis was conducted using G*Power 3.1 to determine the sample size required for the primary outcome. The calculation was based on the comparison of social functioning scores between children with CP and CVI and children with CP without CVI, using an independent-samples *t*-test (means: difference between two independent means [two groups]) from the *t*-test family. The analysis was specified with a large effect size (Cohen’s d = 0.80), statistical power of 80% (1 − β = 0.80), a Type I error probability of 5% (α = 0.05), a two-tailed test, and equal allocation between the groups (1:1). Under these specifications, a minimum of 52 children was required, corresponding to 26 participants in each group. Sample-size adequacy was also examined for the regression analysis. For a fixed multiple regression model comprising seven predictors, the calculation was specified with f^2^ = 0.35, α = 0.05, and statistical power of 80%. These specifications yielded a minimum requirement of 49 participants. Accordingly, the final sample of 70 children exceeded the sample size required for the overall regression model.

Group allocation was based on diagnoses recorded in hospital-issued medical board reports. CVI had been diagnosed by an ophthalmologist following an assessment of visual function, ophthalmological findings, and brain MRI findings. The medical board reports available to the research team recorded the CVI diagnosis but did not provide the detailed findings of these assessments. No additional diagnostic determination or reassessment of CVI was undertaken by the research team. Children aged 4–7 years with both CP and CVI documented in their medical board reports were assigned to the CP and CVI group, whereas those with documented CP but no CVI diagnosis recorded in their medical board reports were assigned to the CP without CVI group. Thus, classification of both groups relied on the same source of clinical documentation. All children included in the study had quadriparetic CP. Children were excluded if they had uncontrolled neurological, orthopedic, or psychiatric conditions, or syndromes other than CP and CVI, that could influence assessment outcomes, or if they were unable to complete the assessments.

A total of 75 children who met the eligibility criteria were enrolled in the study, including 38 children with CP and CVI and 37 children with CP without CVI. During the assessment process, three children from the CP and CVI group and two children from the CP without CVI group were excluded from the final analysis because they did not complete the assessments. Thus, the study was completed with a total of 70 children, comprising 35 children with CP and CVI and 35 children with CP without CVI. None of these children had participated in the previously cited studies [12,13,14,15].

### 2.3. Assessments

#### 2.3.1. Sociodemographic and Clinical Information Form

Children’s sociodemographic characteristics, such as age and sex, as well as clinical characteristics related to CP, were recorded using a sociodemographic and clinical information form developed by the researchers. CVI status was recorded based on official medical board reports issued by the hospital. These data were collected to describe the basic sociodemographic and clinical characteristics of the sample and to compare the characteristics of children with CP and CVI and children with CP without CVI.

Children’s gross motor function levels were classified using the Gross Motor Function Classification System (GMFCS), their ability to handle objects during daily activities using the Manual Ability Classification System (MACS), and their speech performance using the Viking Speech Scale (VSS). Social communication characteristics were assessed using the Social Communication Questionnaire (SCQ), while social functioning was assessed using the Social Function domain of the Pediatric Evaluation of Disability Inventory (PEDI). GMFCS, MACS, and VSS levels were assigned by the same therapist experienced in the assessment of children with CP. As these classifications were performed by a single evaluator, no interrater reliability procedure was applied. The PEDI was administered by the therapist through an interview with the child’s primary caregiver, whereas the SCQ was completed by the same primary caregiver, with assistance from the therapist when needed. Thus, the same caregiver provided information for both caregiver-based assessments for each child. All assessments were conducted by experienced researchers and took approximately 35 min to complete.

#### 2.3.2. Gross Motor Function Classification System (GMFCS)

The GMFCS is a five-level classification system developed by Palisano et al. to classify gross motor function in children with CP [17]. It reflects self-initiated performance in everyday life, particularly in sitting, transfers, and mobility, using age-specific functional descriptions. The classification extends from relatively independent mobility at Level I to severe limitations in independent mobility at Level V, with progressively higher levels reflecting greater gross motor limitations. The validity and reliability of the Turkish version were established by El et al., who reported an interrater reliability of ICC = 0.97, an overall agreement of 89%, and a weighted κ = 0.86. Test–retest reliability was reported as ICC = 0.94 [18].

#### 2.3.3. Manual Ability Classification System (MACS)

The MACS, developed by Eliasson et al., categorizes the usual object-handling performance of children with CP into five levels [19]. It reflects how children typically use their hands to hold, carry, and manipulate objects in everyday activities rather than their maximum manual capacity. The classification ranges from effective object handling at Level I to markedly restricted object handling requiring extensive assistance at Level V, with higher levels representing greater limitations in manual ability. The cross-cultural adaptation, validity, and reliability of the Turkish version of the MACS were established by Akpınar et al. Interrater reliability coefficients for the Turkish version ranged from ICC = 0.89 to 0.96, while test–retest reliability coefficients ranged from ICC = 0.91 to 0.98 [20].

#### 2.3.4. Viking Speech Scale (VSS)

The VSS, developed by Pennington et al., categorizes the usual speech performance of children with CP into four levels according to the impact of motor speech impairment on intelligibility [21]. The classification ranges from intelligible speech without an evident effect of motor impairment at Level I to the absence of understandable speech at Level IV. Higher levels therefore represent progressively greater motor speech impairment and poorer speech intelligibility. The validity and reliability of the Turkish version of the VSS were established by Seyhan-Bıyık et al. in children with CP aged 4–18 years. ICC values for the Turkish version were reported to range from 0.848 to 0.995 [22].

#### 2.3.5. Pediatric Evaluation of Disability Inventory (PEDI)

Haley et al. developed the PEDI to characterize functional performance in children’s everyday activities [23]. The instrument addresses three areas: Self-Care, Mobility, and Social Function. Within the Social Function domain, items concern communication, interaction with others, problem-solving, and performance of socially relevant everyday tasks. The PEDI also includes an evaluation of the assistance provided by caregivers during functional activities. For the purposes of this study, assessment was restricted to the Social Function domain. This domain comprises 65 functional-skill items, with each item assigned a score of 0 when the skill cannot be performed and 1 when it can be performed. Item scores were summed to obtain a raw score between 0 and 65, with increasing scores reflecting better social functioning. We used the raw score because our analyses focused on differences in Social Function skills between the two clinical groups and on the relationships of these skills with the clinical and functional variables examined in the study; comparison with age-based normative performance was not an objective of the analysis. The Turkish PEDI was previously examined by Erkin et al., who reported Cronbach’s α values of at least 0.98 and intraclass correlation coefficients of at least 0.96 [24].

#### 2.3.6. Social Communication Questionnaire (SCQ)

The SCQ, developed by Rutter, Bailey, and Lord, is a caregiver-completed screening measure primarily intended to capture autism-related characteristics of social communication and behavior in children [25]. It includes 40 items addressing reciprocal social interaction, communication, and restricted, repetitive, and stereotyped patterns of behavior. The Turkish Lifetime version was administered in the present study. Each item is scored dichotomously (0 or 1) according to whether the behavior in question is present or absent. Item 1 establishes whether the child uses short phrases or sentences and is excluded from calculation of the total score, resulting in a possible score range of 0–39. SCQ scores were calculated according to the questionnaire’s scoring instructions. Increasing total scores represent a greater presence of the social communication and behavioral characteristics captured by the SCQ; they were not interpreted as indicating primary social communication difficulties. Evidence supporting the Turkish version has been reported in preschool-aged children by Öner et al., with Cronbach’s α of 0.88 for the total score [26], and in children and adolescents aged 4–18 years by Avcil et al., who reported Cronbach’s α of 0.80 and test–retest ICCs between 0.87 and 0.96 [27].

### 2.4. Statistical Analysis

Data analysis was carried out with IBM SPSS Statistics version 25.0 (IBM Corp., Armonk, NY, USA). Descriptive results for categorical variables are given as frequencies and percentages [n (%)], while continuous variables are presented as mean (SD). Before comparative analyses, the distribution of continuous data was examined within each study group using the Shapiro–Wilk test. No observations were missing for any of the variables analyzed in the 70 participants, and consequently no imputation or other missing-data procedure was applied.

Group comparisons were selected according to variable type. Continuous variables were analyzed with independent-samples *t*-tests, and Cohen’s d was calculated to describe the magnitude of between-group differences. Cohen’s d values of 0.20, 0.50, and 0.80 were taken to indicate small, medium, and large effects, respectively [28]. Analyses of individual SCQ subdomains were regarded as secondary/exploratory; because multiple subdomain comparisons were undertaken, these findings were interpreted cautiously. Categorical distributions were examined with Pearson’s chi-square (χ^2^) test. Their effect sizes were expressed as Cramer’s V, for which 0.10, 0.30, and 0.50 represented small, medium, and large effects, respectively [28]. Spearman’s rank correlations were calculated for PEDI Social Function, SCQ total score, age, GMFCS, MACS, VSS, and CVI status to characterize the relationships among these variables.

The independent associations with social functioning were subsequently examined using hierarchical multiple linear regression. PEDI Social Function score constituted the dependent variable, and predictors were introduced in five successive models. The initial model contained age and sex. CVI status was introduced at the second stage, followed by GMFCS and MACS levels at the third, VSS level at the fourth, and SCQ total score at the fifth and final stage. The sequence of entry was established a priori to examine the contribution of demographic characteristics first, followed successively by CVI status and motor, manual, speech, and social communication characteristics.

Changes in model explanatory performance across the five stages were characterized by R^2^, adjusted R^2^, ΔR^2^, and ΔF. For individual predictors, B, SE, standardized β, and *p*-values are reported. Possible multicollinearity was evaluated from tolerance and variance inflation factor (VIF) statistics; tolerance values above 0.20 together with VIF values below 5 were regarded as acceptable [29]. Because the final model yielded a notably high R^2^, an additional bootstrap procedure was undertaken to examine the stability of its coefficient estimates. This analysis used 1000 resamples and generated 95% bias-corrected and accelerated (BCa) confidence intervals. Statistical testing was two-tailed throughout, with *p* < 0.05 defining statistical significance.

## 3. Results

A total of 70 children aged 48–84 months were included in the final analysis, comprising 35 children with CP and CVI and 35 children with CP without CVI. There were no statistically significant between-group differences in age (*p* = 0.595) or sex distribution (*p* = 0.810) (Table 1).

Statistically significant differences were found between the groups in the distributions of GMFCS (*p* = 0.007), MACS (*p* = 0.043), and VSS (*p* = 0.027) levels. In the CP and CVI group, higher proportions of children were classified at GMFCS Levels III–V, MACS Levels III–V, and VSS Levels III–IV, whereas in the CP without CVI group, higher proportions of children were classified at lower levels, representing better gross motor function, manual ability, and speech performance (Table 2).

Social functioning assessed using the PEDI was significantly lower in children with CP and CVI than in children with CP without CVI (*p* < 0.001). Similarly, children with CP and CVI had higher SCQ total scores than children with CP without CVI (*p* < 0.001). Phrase speech was not used by 4 of 35 children in the CP and CVI group and 3 of 35 children in the CP without CVI group. In secondary/exploratory analyses of the SCQ subdomains, children with CP and CVI also had higher scores in reciprocal social interaction, communication, and restricted, repetitive, and stereotyped behaviors (all *p* < 0.001) (Table 3).

Spearman correlation analysis showed that PEDI Social Function scores were positively correlated with age (ρ = 0.578, *p* < 0.001) and negatively correlated with CVI status (ρ = −0.648, *p* < 0.001), GMFCS (ρ = −0.859, *p* < 0.001), MACS (ρ = −0.831, *p* < 0.001), VSS (ρ = −0.824, *p* < 0.001), and SCQ total scores (ρ = −0.911, *p* < 0.001). Positive correlations were also observed among GMFCS, MACS, and VSS (all *p* < 0.001). The complete correlation matrix is presented in Table 4.

In the hierarchical multiple regression analysis, the first model, which included age and sex, explained 33.9% of the variance in social functioning (R^2^ = 0.339; *p* < 0.001). The addition of CVI status increased the explained variance to 71.3%, accounting for an additional 37.4% of the variance (ΔR^2^ = 0.374; *p* < 0.001). After the addition of GMFCS and MACS levels, the explained variance increased to 92.5% (ΔR^2^ = 0.213; *p* < 0.001). The addition of VSS level accounted for a further 2.2% of the variance (ΔR^2^ = 0.022; *p* < 0.001), while the addition of the SCQ total score in the final step accounted for an additional 2.0% (ΔR^2^ = 0.020; *p* < 0.001). The final model explained 96.7% of the variance in social functioning assessed using the PEDI (R^2^ = 0.967; adjusted R^2^ = 0.963; *p* < 0.001) (Table 5).

In the final regression model, age (β = 0.089; *p* = 0.008), CVI status (β = −0.295; *p* < 0.001), GMFCS level (β = −0.270; *p* < 0.001), VSS level (β = −0.211; *p* < 0.001), and SCQ total score (β = −0.272; *p* < 0.001) were independently associated with social functioning assessed using the PEDI. The presence of CVI, higher GMFCS and VSS levels, and higher SCQ total scores were associated with lower social functioning. In contrast, sex (*p* = 0.106) and MACS level (*p* = 0.061) were not independently associated with social functioning in the final model (Table 6).

To further assess the stability of the regression coefficient estimates, bootstrap results based on 1000 resamples were examined. In the final model, the 95% BCa confidence intervals for CVI status (B = −7.801, 95% BCa CI: −11.368 to −6.226), GMFCS (B = −2.565, 95% BCa CI: −3.538 to −1.648), VSS (B = −2.626, 95% BCa CI: −3.930 to −1.385), and SCQ total score (B = −0.424, 95% BCa CI: −0.573 to −0.142) did not include zero. MACS was not statistically significant based on the bootstrap test (*p* = 0.060), while the bootstrap result for age (*p* = 0.094) differed from the conventional regression result. These findings generally supported the stability of the principal regression coefficient estimates, although the age effect should be interpreted cautiously.

## 4. Discussion

The findings revealed clear differences in functional and social characteristics according to CVI status. Compared with children with CP without CVI, those with CP and CVI showed greater limitations in gross motor function, manual ability, and speech performance, together with lower social functioning and higher SCQ total scores. The final hierarchical model accounted for 96.7% of the variance in social functioning. After all variables were considered simultaneously, higher social functioning was associated with older age, whereas lower social functioning was associated with CVI status, greater gross motor limitations, poorer speech performance, and higher SCQ total scores. Sex and manual ability did not retain statistically significant independent associations in the final model.

Visual function in children with CP has previously been shown to be associated with motor, manual, and communication functions. Baranello et al., using the Visual Function Classification System (VFCS), developed to classify the visual abilities of children with CP in the context of daily life, demonstrated that visual function represents an important functional domain reflecting the extent and frequency with which a child uses vision during activities and the need for environmental adaptations and support [30]. In the validity study of the Turkish version of the VFCS, visual function was evaluated alongside the GMFCS, MACS, and Communication Function Classification System (CFCS), supporting associations between visual function and gross motor, manual, and communication functions [31]. Similarly, studies evaluating different functional classification systems together have reported associations between VFCS levels and GMFCS, MACS, and CFCS levels [32,33]. Consistent with this literature, our study showed that children with CP and CVI were more frequently classified at higher GMFCS, MACS, and VSS levels than children with CP without CVI. Taken together, these findings indicate that CVI in children with CP may occur alongside limitations across several functional domains, including gross motor function, manual ability, and speech performance. Because the present study was cross-sectional, however, the observed differences cannot establish a causal effect of CVI on these functions. Instead, visual, motor, manual, and speech characteristics may reflect different aspects of the underlying neurological involvement in CP.

The presence of visual impairment in children with CP has been considered in relation to a range of functional domains beyond motor abilities, including sensory, behavioral, communication, and daily life functioning. In a recent study specifically focusing on CVI, Collart et al. reported that functional vision difficulties were associated with lower health-related quality of life in children with CVI [34]. Focusing more directly on the social domain, Kılıç et al. showed that children with CP and CVI had lower social competence than children with CP without CVI and typically developing children [15]. In our study, children with CP and CVI also had significantly lower social functioning than children with CP without CVI, with a large effect size. Furthermore, children with CP and CVI had higher scores across all SCQ domains, including reciprocal social interaction, communication, and restricted/repetitive behaviors. This pattern indicates that the between-group differences were not limited to social competence but were also evident across several SCQ-assessed social communication and behavioral characteristics relevant to everyday social functioning. However, these between-group differences should be interpreted in light of differences in functional severity and unmeasured clinical characteristics; they cannot be attributed to CVI alone.

Social functioning in children with CP has previously been reported to be associated with developmental, motor, and communication characteristics. Burgess et al. showed that social function skills develop with age and vary according to communication function level [9], while motor and communication abilities have also been reported to be associated with subsequent social functioning [35]. Regarding visual function, functional vision difficulties in children with CVI have been associated with daily life-related outcomes [34] and lower social competence has been reported in children with CP and CVI [15]. In our hierarchical regression analysis, age and sex initially explained 33.9% of the variance in social functioning, while the addition of CVI status accounted for an additional 37.4% of the variance. The addition of gross motor function and manual ability accounted for a further 21.3%, followed by speech performance with 2.2% and SCQ total score with 2.0%. In the final model, older age was independently associated with higher social functioning in the conventional regression analysis, whereas CVI status, greater limitations in gross motor function, poorer speech performance, and higher SCQ total scores were independently associated with lower social functioning; sex and manual ability did not retain independent associations. However, the association with age did not remain statistically significant in the bootstrap analysis and should therefore be interpreted cautiously. The 37.4% increase in explained variance observed when CVI status was added after age and sex should be interpreted in the context of the hierarchical entry order and does not represent the unique contribution of CVI. Nevertheless, CVI status remained independently associated with social functioning in the final model after the other included variables were considered. At the same time, the independent associations of gross motor function, speech performance, and SCQ total score support the multidimensional nature of social functioning in relation to visual, motor, communication, and developmental characteristics.

CVI encompasses different visual difficulties that may affect everyday social interactions in different ways. For example, difficulties recognizing faces or locating relevant information in visually complex settings could make it harder for a child to recognize social partners and follow interactions [3,36]. These possibilities offer clinical context for the group differences observed in social functioning and SCQ scores. However, we did not assess these specific visual difficulties in individual children and therefore cannot determine whether any particular CVI-related difficulty contributed to the observed differences.

This study has some limitations. First, the study was conducted at a single rehabilitation center, which may limit the generalizability of the findings to populations of children with CP with different clinical and sociodemographic characteristics. Second, although social functioning and social communication were assessed, family, school, and environmental characteristics that may be associated with children’s social functioning were not included in the assessment. Third, although CVI status was based on clinically established diagnoses documented in official medical board reports, functional vision was not additionally characterized using a standardized classification system such as the VFCS, and specific CVI-related visual difficulties were not separately assessed. Therefore, the study could not provide a detailed characterization of functional vision levels and specific visual difficulties within the CVI group or examine social functioning according to different CVI-related visual profiles. Fourth, although GMFCS, MACS, and VSS levels were included in the hierarchical regression model, cognitive functioning and CFCS levels were not assessed. Therefore, the potential influence of these unmeasured clinical characteristics on social functioning could not be accounted for, and residual confounding cannot be excluded. Fifth, GMFCS, MACS, and VSS are ordinal classification systems and were entered into the regression model as numeric levels. This approach assumes equal distances between adjacent classification levels; therefore, the corresponding regression coefficients should be interpreted with caution. Sixth, raw rather than scaled or normative PEDI Social Function scores were used. Therefore, the findings reflect functional skill performance within the study sample and should not be interpreted as indicating children’s age-referenced or normative standing relative to the general population. Seventh, although bootstrap analysis was used to examine the stability of the regression coefficient estimates, the notably high R^2^ of the final model should be interpreted cautiously because the model was not externally validated or cross-validated in an independent sample. Future multicenter studies incorporating these contextual factors and standardized functional vision assessments may contribute to a more comprehensive understanding of social functioning in children with CP.

## 5. Conclusions

In this study, children with CP and CVI showed greater limitations in gross motor function, manual ability, and speech performance, as well as lower social functioning and higher scores for SCQ-assessed social communication and behavioral characteristics than children with CP without CVI. In addition, the presence of CVI, greater limitations in gross motor function, poorer speech performance, and higher SCQ total scores were independently associated with lower social functioning. These findings highlight the importance of assessing speech performance, SCQ-assessed social communication and behavioral characteristics, and social functioning in daily life, in addition to motor and visual functions, as part of the routine evaluation of children with CP. Future quantitative studies should investigate the independent associations of additional variables, such as cognitive function, sensory processing, behavioral characteristics, and environmental factors, with social functioning by incorporating them into regression models. Qualitative studies exploring children’s social experiences in greater depth, as well as intervention studies aimed at improving social functioning, are also warranted.

## Figures and Tables

**Table 1:** Sociodemographic characteristics of the study groups.

Characteristics	Children with CP and CVI (*n* = 35)	Children with CP Without CVI (*n* = 35)	*p*	Effect Size
	Mean (SD)	Mean (SD)		
**Age (months)**	65.49 (10.61)	66.86 (10.89)	0.595 ^a^	0.13 ^c^
**Sex**	***n*** **(%)**	***n*** **(%)**		
Female	19 (54.3)	20 (57.1)	0.810 ^b^	0.03 ^d^
Male	16 (45.7)	15 (42.9)		

CP: Cerebral palsy; CVI: cerebral visual impairment; SD: standard deviation; n: number of participants; %: percentage. ^a^ Independent-samples *t*-test. ^b^ Pearson’s chi-square test. ^c^ Cohen’s d. ^d^ Cramer’s V.

**Table 2:** Distribution of GMFCS, MACS, and VSS levels in the study groups.

Classification	Children with CP and CVI (*n* = 35)	Children with CP Without CVI (*n* = 35)	*p*	Effect Size
	*n* (%)	*n* (%)		
**GMFCS**			**0.007 ^a^**	**0.45 ^b^**
Level I	4 (11.4)	9 (25.7)		
Level II	3 (8.6)	12 (34.3)		
Level III	10 (28.6)	6 (17.1)		
Level IV	10 (28.6)	2 (5.7)		
Level V	8 (22.9)	6 (17.1)		
**MACS**			**0.043 ^a^**	**0.38 ^b^**
Level I	5 (14.3)	12 (34.3)		
Level II	4 (11.4)	10 (28.6)		
Level III	12 (34.3)	6 (17.1)		
Level IV	7 (20.0)	4 (11.4)		
Level V	7 (20.0)	3 (8.6)		
**VSS**			**0.027 ^a^**	**0.36 ^b^**
Level I	7 (20.0)	11 (31.4)		
Level II	6 (17.1)	14 (40.0)		
Level III	12 (34.3)	7 (20.0)		
Level IV	10 (28.6)	3 (8.6)		

CP: Cerebral palsy; CVI: cerebral visual impairment; GMFCS: Gross Motor Function Classification System; MACS: Manual Ability Classification System; VSS: Viking Speech Scale; n: number of participants; %: percentage. ^a^ Pearson’s chi-square test. ^b^ Cramer’s V.

**Table 3:** Comparison of PEDI Social Function and SCQ scores between the study groups.

Variables	Children with CP and CVI (*n* = 35)	Children with CP Without CVI (*n* = 35)	*p*	Effect Size
	Mean (SD)	Mean (SD)		
**PEDI Social Function**	27.94 (11.09)	45.09 (9.20)	<0.001 ^a^	1.68 ^b^
**SCQ Total Score**	25.23 (6.72)	14.80 (6.82)	<0.001 ^a^	1.54 ^b^
SCQ-Reciprocal Social Interaction	10.37 (3.26)	5.80 (2.87)	<0.001 ^a^	1.49 ^b^
SCQ-Communication	8.40 (2.32)	4.89 (2.63)	<0.001 ^a^	1.42 ^b^
SCQ-Restricted, Repetitive, and Stereotyped Behaviors	4.86 (1.91)	2.23 (1.48)	<0.001 ^a^	1.54 ^b^

CP: Cerebral palsy; CVI: cerebral visual impairment; PEDI: Pediatric Evaluation of Disability Inventory; SCQ: Social Communication Questionnaire; SD: standard deviation. ^a^ Independent-samples *t*-test. ^b^ Cohen’s *d*.

**Table 4:** Spearman Correlation Matrix Among Study Variables.

	Overall Sample: Children with CP with and Without CVI (*n* = 70)						
Variable	Age	CVI Status	GMFCS	MACS	VSS	SCQ Total Score	PEDI Social Function
**Age**	—						
**CVI status**	−0.063	—					
**GMFCS**	−0.570 ***	0.327 **	—				
**MACS**	−0.640 ***	0.333 **	0.834 ***	—			
**VSS**	−0.643 ***	0.309 **	0.785 ***	0.768 ***	—		
**SCQ total score**	−0.443 ***	0.642 ***	0.744 ***	0.689 ***	0.718 ***	—	
**PEDI Social Function**	0.578 ***	−0.648 ***	−0.859 ***	−0.831 ***	−0.824 ***	−0.911 ***	—

Note: Values are Spearman’s rank correlation coefficients (ρ). CVI: Cerebral visual impairment; GMFCS: Gross Motor Function Classification System; MACS: Manual Ability Classification System; VSS: Viking Speech Scale; SCQ: Social Communication Questionnaire; PEDI: Pediatric Evaluation of Disability Inventory. CVI status: 1 = without CVI, 2 = with CVI. ** *p* < 0.01; *** *p* < 0.001.

**Table 5:** Hierarchical multiple linear regression analysis for social functioning.

	Overall Sample: Children with CP with and Without CVI (*n* = 70)						
Model	R^2^	Adjusted R^2^	SE of Estimate	ΔR^2^	ΔF	Model F	*p*
Model 1	0.339	0.319	10.969	0.339	17.196	17.196	<0.001
Model 2	0.713	0.700	7.285	0.374	85.894	54.637	<0.001
Model 3	0.925	0.920	3.771	0.213	91.141	158.409	<0.001
Model 4	0.947	0.942	3.190	0.022	26.423	187.307	<0.001
Model 5	0.967	0.963	2.546	0.020	36.895	257.043	<0.001

R^2^: Coefficient of determination; SE: standard error; ΔR^2^: change in R^2^; ΔF: F-change; CP: cerebral palsy; CVI: cerebral visual impairment; Dependent variable: PEDI Social Function score; *p* < 0.05. ^a^ Model 1: Age and sex. ^b^ Model 2: Age, sex, and CVI status. ^c^ Model 3: Age, sex, CVI status, GMFCS, and MACS. ^d^ Model 4: Age, sex, CVI status, GMFCS, MACS, and VSS. ^e^ Model 5: Age, sex, CVI status, GMFCS, MACS, VSS, and SCQ total score.

**Table 6:** Hierarchical multiple regression coefficients for predictors of social functioning.

	Children with CP with and without CVI (*n* = 70)							
Model	Predictor	B	SE	β	t	*p*	Tolerance	VIF
**Model 1**	Constant	−7.644	9.179	—	−0.833	0.408	—	—
	Age	0.715	0.124	0.575	5.788	<0.001	1.000	1.000
	Sex	−2.184	2.640	−0.082	−0.827	0.411	1.000	1.000
**Model 2**	Constant	19.209	6.750	—	2.846	0.006	—	—
	Age	0.666	0.082	0.536	8.104	<0.001	0.996	1.005
	Sex	−1.736	1.754	−0.065	−0.990	0.326	0.999	1.001
	CVI status	−16.180	1.746	−0.613	−9.268	<0.001	0.995	1.005
**Model 3**	Constant	59.188	4.729	—	12.515	<0.001	—	—
	Age	0.201	0.057	0.161	3.513	0.001	0.553	1.808
	Sex	−0.453	0.914	−0.017	−0.495	0.622	0.987	1.014
	CVI status	−11.300	0.981	−0.428	−11.522	<0.001	0.845	1.183
	GMFCS	−4.592	0.571	−0.483	−8.045	<0.001	0.323	3.092
	MACS	−1.680	0.632	−0.173	−2.657	0.010	0.276	3.623
**Model 4**	Constant	67.639	4.326	—	15.637	<0.001	—	—
	Age	0.116	0.051	0.094	2.281	0.026	0.496	2.017
	Sex	−0.938	0.779	−0.035	−1.204	0.233	0.972	1.029
	CVI status	−10.698	0.838	−0.405	−12.768	<0.001	0.829	1.207
	GMFCS	−3.485	0.529	−0.367	−6.593	<0.001	0.270	3.706
	MACS	−1.086	0.547	−0.112	−1.984	0.052	0.264	3.793
	VSS	−3.362	0.654	−0.270	−5.140	<0.001	0.303	3.303
**Model 5**	Constant	67.048	3.454	—	19.413	<0.001	—	—
	Age	0.111	0.041	0.089	2.730	0.008	0.496	2.018
	Sex	−1.019	0.622	−0.038	−1.640	0.106	0.972	1.029
	CVI status	−7.801	0.821	−0.295	−9.497	<0.001	0.549	1.821
	GMFCS	−2.565	0.448	−0.270	−5.722	<0.001	0.239	4.184
	MACS	−0.838	0.439	−0.086	−1.910	0.061	0.261	3.826
	VSS	−2.626	0.536	−0.211	−4.899	<0.001	0.287	3.481
	SCQ total score	−0.424	0.070	−0.272	−6.074	<0.001	0.265	3.770

B: Unstandardized regression coefficient; SE: standard error; β: standardized regression coefficient; CVI: cerebral visual impairment; GMFCS: Gross Motor Function Classification System; MACS: Manual Ability Classification System; VSS: Viking Speech Scale; SCQ: Social Communication Questionnaire; VIF: variance inflation factor. Sex: 1 = female, 2 = male; CVI status: 1 = without CVI, 2 = with CVI; GMFCS: I = 1 to V = 5; MACS: I = 1 to V = 5; VSS: I = 1 to IV = 4. Dependent variable: PEDI Social Function score; *p* < 0.05. a. Model 1: Age and sex. b. Model 2: Age, sex, and CVI status. c. Model 3: Age, sex, CVI status, GMFCS, and MACS. d. Model 4: Age, sex, CVI status, GMFCS, MACS, and VSS. e. Model 5: Age, sex, CVI status, GMFCS, MACS, VSS, and SCQ total score.

## Data Availability

The data presented in this study are available from the corresponding author upon reasonable request. The data are not publicly available due to privacy and ethical considerations.

## Supplementary Materials

The following supporting information can be downloaded at https://www.mdpi.com/article/10.3390/children13101310/s1. File S1: STROBE Statement—checklist of items that should be included in reports of cross-sectional studies.
